# Trait Self-Control, Identified-Introjected Religiosity and Health-Related-Feelings in Healthy Muslims: A Structural Equation Model Analysis

**DOI:** 10.1371/journal.pone.0126193

**Published:** 2015-05-11

**Authors:** Walid Briki, Asma Aloui, Nicola Luigi Bragazzi, Anis Chaouachi, Thomas Patrick, Karim Chamari

**Affiliations:** 1 University of French West Indies, Department of Sport Sciences, ACTES Laboratory, Pointe-à-Pitre, Guadeloupe, France; 2 Qatar University, College of Arts and Sciences, Sport Science Program, Doha, Qatar; 3 Sport Performance Optimization Laboratory, National Center of Medicine and Sciences in Sport (CNMSS), Tunis, Tunisia; 4 High Institute of Sport and Physical Education, Gafsa University, Gafsa, Tunisia; 5 School of Public Health, Department of Health Sciences (DISSAL), Genoa University, Genoa, Italy; 6 Department of Neuroscience, Rehabilitation, Ophthalmology, Genetics, Maternal and Child Health (DINOGMI), Section of Psychiatry, Genoa University, Genoa, Italy; 7 Italian Islamic University, Lecce, Italy; 8 National Sports Medicine Program, ASPETAR Orthopedic & Sports Medicine Hospital, Doha, Qatar; 9 Sport Performance Research Institute of New Zealand (SPRINZ), Auckland, New Zealand; 10 Athlete Health and Performance Research Center, ASPETAR Orthopedic & Sports Medicine Hospital, Doha, Qatar; Saarland University, GERMANY

## Abstract

**Aim:**

The present study attempted to test McCullough and Willoughby’s hypothesis that self-control mediates the relationships between religiosity and psychosocial outcomes. Specifically, this study examined whether trait self-control (TSC) mediates the relationship of identified-introjected religiosity with positive and negative health-related-feelings (HRF) in healthy Muslims.

**Methods:**

Two hundred eleven French-speaking participants (116 females, 95 males; *M_age_* = 28.15, *SD_age_* = 6.90) answered questionnaires. One hundred ninety participants were retained for the analyses because they reported to be healthy (105 females, 85 males; *M_age_* = 27.72, *SD_age_* = 6.80). To examine the relationships between religiosity, TSC and HRF, two competing mediation models were tested using structural equation model analysis: While a starting model used TSC as mediator of the religiosity-HRF relationship, an alternative model used religiosity as mediator of the TSC-HRF relationship.

**Results:**

The findings revealed that TSC mediated the relationship between identified religiosity and positive HRF, and that identified religiosity mediated the relationship between TSC and positive and negative HRF, thereby validating both models. Moreover, the comparison of both models showed that the starting model explained 13.211% of the variance (goodness of fit = 1.000), whereas the alternative model explained 6.877% of the variance (goodness of fit = 0.987).

**Conclusion:**

These results show that the starting model is the most effective model to account for the relationships between religiosity, TSC, and HRF. Therefore, this study provides initial insights into how religiosity influences psychological health through TSC. Important practical implications for the religious education are suggested.

## Introduction

Religion, defined as “…cognition, affect, and behavior that arise from awareness of, or perceived interaction with, supernatural entities that are presumed to play an important role in human affairs” [1], is a psychosocial force capable of heavily impacting and modifying trajectories of human lives. The literature of religion psychology reveals that adherence to religion is generally beneficial for psychological health (for review, see [1]). However, the mechanisms underlying this positive relationship between religion and psychological health still need to be explored. The present study consists in examining whether *self-control*―representing a crucial construct of self-regulation―mediates the links between religiosity and health-related-outcomes. Self-regulation is defined as “…the process by which a system uses information about its present state to change that state.” ([1], p. 71). Self-control refers to the people’s capacity “…to counteract or override a prepotent response”, so that “…when people exert self-control, they modify their response tendencies in a fashion that involves suppressing one goal so as to pursue another one that is judged to have greater long-term utility” ([1], p. 72).

### Religiosity and Psychosocial Outcomes

Globally, research revealed that global religiousness, defined as the adherence to a religion that involves predefined behaviors and rituals (e.g., [1]), was positively related to positive psychosocial outcomes, and negatively related to negative psychosocial outcomes. Specifically, it was found that global religiousness was related to higher levels of longevity [2], satisfaction with life (i.e., wellbeing) [3–7], marital satisfaction [8], health behaviors (e.g., higher frequency of visiting physicians, [9]), academic performance (e.g., [10]), and lower levels of mortality [11], anxiety [5, 12], depression [12, 13], divorce [8], and criminality [14]. Research also examined the link between religious motivation (i.e., the way religious people commit to the religion) and psychosocial outcomes, and found that intrinsic religious motivation, corresponding to the use of religion as one’s ultimate motivation (e.g., “I enjoy to spend time in private thought and prayer”), was negatively related to depression. It was also shown that extrinsic religious motivation, defined as the use of religion as a means to reach a specific goal (e.g., “I pray for wellbeing”), was positively associated with depression [12]. Moreover, examining the psychosocial consequences of religious coping (i.e., the way religious people deal with stressful events), research reported that positive religious coping was positively related to wellbeing [6], and negatively related to anxiety and depression [12, 6]. Positive religious coping corresponds to a strategy consisting in looking for a stronger connection with God when facing a problem in life. Conversely, it was found that negative religious coping, defined as a strategy consisting in attributing one’s problem to God’s punishment, was positively related to anxiety and depression [12, 6], but unrelated to wellbeing [6].

As a result, research has reported that global religiousness, religious motivation, and religious coping might positively or negatively influence affects, cognitions, and behaviors. Specifically, it appeared that global religiousness, intrinsic religious motivation, and positive religious coping might be related to adaptive psychological implications, while extrinsic religious motivation and negative religious coping might be related or unrelated to maladaptive psychological implications. This suggests that global religiousness, intrinsic religious motivation, and positive religious coping would be personality patterns reflecting a positive internalization of religion, called *identified religiosity* [1, 15]. By contrast, extrinsic religious motivation and negative religious coping would reflect a negative internalization of religion, called *introjected religiosity* [1, 15]. Interestingly, authors who examined the links between identified-introjected religiosity and psychological outcomes observed that identified religiosity was related to higher levels of self-esteem (i.e., assessment of one’s own worth) and wellbeing, and lower levels of anxiety, depression, and social dysfunction, and that introjected religiosity was related to higher levels of anxiety, interpersonal conflicts, and lower levels of self-esteem [16, 17].

### The Mediating Effect of Self-Control

Based on Carver and Scheier’s [18, 19] control-process model of self-regulation, McCullough and Willoughby [1] proposed a model intended to explain the associations between religiosity and its psychosocial outcomes in religious people. McCullough and Willoughby [1] posits that religiosity may affect psychosocial outcomes *through* self-regulation and self-control. Self-regulation corresponds to self-corrective adjustments that serve the goal pursued and its effectiveness requires the ability of pursuing clear goals [18, 19]. The model posits that the way of people internalize religion influences the goals selection and their importance for the self in such a way that goal systems hierarchically organize from the most abstract goals (e.g., *a concept system*: being a good Muslim; *a principle*: being respectful, responsible, polite, self-disciplined) to the most concrete goals (e.g., *a program*: distract oneself from hostile thoughts; *a sequence*: reading Quran).

From this perspective, a strong adherence to religion would lead people both to embrace relevant goals with regard to the religious sphere (e.g., being responsible) and eschew other goals (e.g., pursuing pleasure, being independent), thus leading to activate monitoring that corresponds to “…a state of self-awareness about how one is behaving relative to a norm or standard” ([20], p. 1). Testing this hypothesis, Carter and colleagues [20] reported that global religiousness predicted monitoring, and that monitoring mediated the link between global religiousness and self-control. According to McCullough and Willoughby [1], self-control would be the key variable of self-regulation of affects and behaviors in religious people, in the sense that the effect of religiosity on psychosocial benefits would be mediated by self-control. Authors evidenced this hypothesis by reporting findings displaying that self-control partially mediated the link of global religiousness with criminality [1] and substance-use behaviors [21–23]. In the same vein, other studies showed that individuals who were implicitly exposed to religious themes, relative to those who were implicitly exposed to non-religious themes, behaved more generously [24], more honestly [25, 26], and exercised greater self-control [27]. Finally, consistent with the McCullough and Willoughby’s model, these results suggest that religiosity enhances self-control, which ultimately influences moral behaviors and psychosocial outcomes.

### Overview

Because no study, to date, examined whether self-control may mediate the link between religiosity and health-related-feelings (HRF), the present study attempted to examine whether trait self-control (TSC) mediates the relationships between identified-introjected religiosity and HRF (assessed through different subscales, such as wellbeing, general self-esteem, affects, anxiety, and depression) in healthy Muslims. Identified religiosity was assessed through global religiousness, intrinsic religious motivation, and positive religious coping, while introjected religiosity was assessed through extrinsic religious motivation and negative religious coping. Concerning the hypotheses, consistent with previous findings, it was expected that identified and introjected religiosity would be linked to HRF through TSC. Specifically, within the mediations, it was expected that the identified religiosity and TSC would be positively related to positive HRF and negatively related to negative HRF. It was also expected that introjected religiosity would be positively related to negative HRF and negatively related to positive HRF. Moreover, to test the effectiveness of these hypotheses, we compared two competing models, where the starting model used TSC as mediator of the relationship between religiosity and HRF, whereas the alternative model used religiosity as mediator of the relationship between TSC and HRF. To compare both models, we used a structural equation model (SEM) analysis.

## Methods

### Participants

Two hundred eleven French-speaking participants (116 females, 54.98%; 95 males, 45.02%; *M*_age_ = 28.15, *SD*_age_ = 6.90) were recruited through Islamic schools, cultural associations, mosques, Islamic conferences, and universities, in May/June 2013 (thus outside the Ramadan period); they volunteered to participate in the study. They came from non-Muslim countries (i.e., France, Belgium, Luxembourg, Canada, USA, Switzerland, Netherlands, and Russia; *n* = 119, 56.40%; 70 females, 58.82%; 49 males, 41.18%) and Muslim countries (i.e., Tunisia, Algeria, Morocco, Saudi Arabia, Brunei, UAE, and Qatar; *n* = 92; 46 females, 50.00%, 46 males, 50.00%). They were all Sunni Muslims.

One hundred ninety participants (105 females, 85 males; *M*_age_ = 27.72, *SD*_age_ = 6.80), coming from non-Muslim countries (i.e., France, Belgium, Luxembourg, Canada, USA, Switzerland, and Russia; *n* = 104; 62 females, 42 males) and Muslim countries (i.e., Tunisia, Algeria, Morocco, Saudi Arabia, Brunei, UAE, and Qatar; *n* = 86; 43 females, 43 males), were retained for analyses because they reported that they had no current psychiatric and/or somatic illness. Removing participants who reported having current psychiatric (*n* = 16, 76.19%), somatic illness (*n* = 3, 14.29%), or both kinds of illness (*n* = 2, 9.52%) enabled us to focus only on *healthy* people. As a result, the present study was interested in examining the psychological functioning of healthy Sunni Muslims. This sample included participants who were heterogeneous on their professional status (i.e., *working*: *n* = 168, 88.42%, or *not working*: *n* = 22, 11.58%) and their relationship status (i.e., *with a partner*: *n* = 59, 31.05%, or *without a partner*: *n* = 131, 68.95%).

### Study Design

The study protocol has been approved by the clinical research ethics committee of the National Center of Medicine and Sciences in Sport of Tunis before the commencement of the survey. Informed written consent has been obtained from each participant. Requests for data from the present study should be directed to the first author of this article. This study was completed in one month (in May/June 2013). Its setup included a form that was accessible to participants via a specific web address. This form included general information about the study, a consent form, and questions. Before answering questions, participants were ensured that their responses would remain confidential. Then, they were asked to enter a pseudonym on the website (to preserve their anonymity) and were invited to pursue the study in answering questions.

### Measures

#### Personality patterns

The 7-item positive religious coping subscale (e.g., “When I face a problem in life, I look for a stronger connection with Allah”) (α = .83) and the 3-item negative religious coping subscale (e.g., “When I face a problem in life, I believe that I am being punished by Allah for bad actions I did”) (α = .84) of the Psychological Measure Islamic Religiousness (PMIR; [28]) were measured (1 = “*strongly disagree*”; 9 = “*strongly agree*”). The 2-item global religiousness subscale of PMIR (i.e., “How do you describe your religiousness?” and “How do you describe your spirituality?”) (α = .53) was also measured (1 = “*very low*”; 9 = “*very high*”). The Religious Orientation Scale-Revised questionnaire (ROSR; [29]) was adapted to Islam context to measure the 8-item intrinsic motivation subscale (e.g., “I enjoy reading about my religion”) (α = .72), the 3-item extrinsic personal motivation subscale (e.g., “I pray mainly to gain relief and protection”) (α = .59), and the 3-item extrinsic social motivation subscale (e.g., I go to mosque because it helps me to make friends”) (α = .77). The items of ROSR were scored from 1 (“*strongly disagree*”) to 9 (“*strongly agree*”). TSC was measured using the 13-item scale provided by Tangney, Baumeister, and Boone [30] (e.g., “I am good at resisting temptation”; 1 = “*not at all*”, 9 = “*very much so*”) (α = .74).

#### HRF

The 5-item positive affect scale (e.g., “I feel inspired”) (α = .78) and the 5-item negative affect scale (e.g., “I feel hostile”) (α = .76) of the International Positive and Negative Affect Schedule Short Form [31], the 5-item general self-esteem scale of the Physical Self Inventory-25 [32] (e.g., “I have a good opinion of myself”) (α = .69), and the 10-item State Self-control Capacity Scale [33] (e.g., “I can’t absorb any information” [reverse-coded item]) (α = .81) were used to measure positive and negative affect, general self-esteem, and state self-control, respectively. Depression was measured by using the 4-item Center for Epidemiological Studies—Depression—Visual Analogical Scale [34] (e.g., “I have crying spells or feel like it”) (α = .65). Cognitive anxiety was assessed by a single item of the State—Trait Anxiety Inventory [35] (“I am worried”). All the previous items were scored from 1 (“*not at all*”) to 9 (“*very much so*”). Wellbeing was measured by the “optional” item of the Personal Wellbeing Index—Adult [36] (“Thinking about your own life and personal circumstances, how satisfied are you with your life as a whole?”) (1 = “*completely dissatisfied*”; 9 = “*completely satisfied*”).

### Statistical Analysis

**Fig 1:**
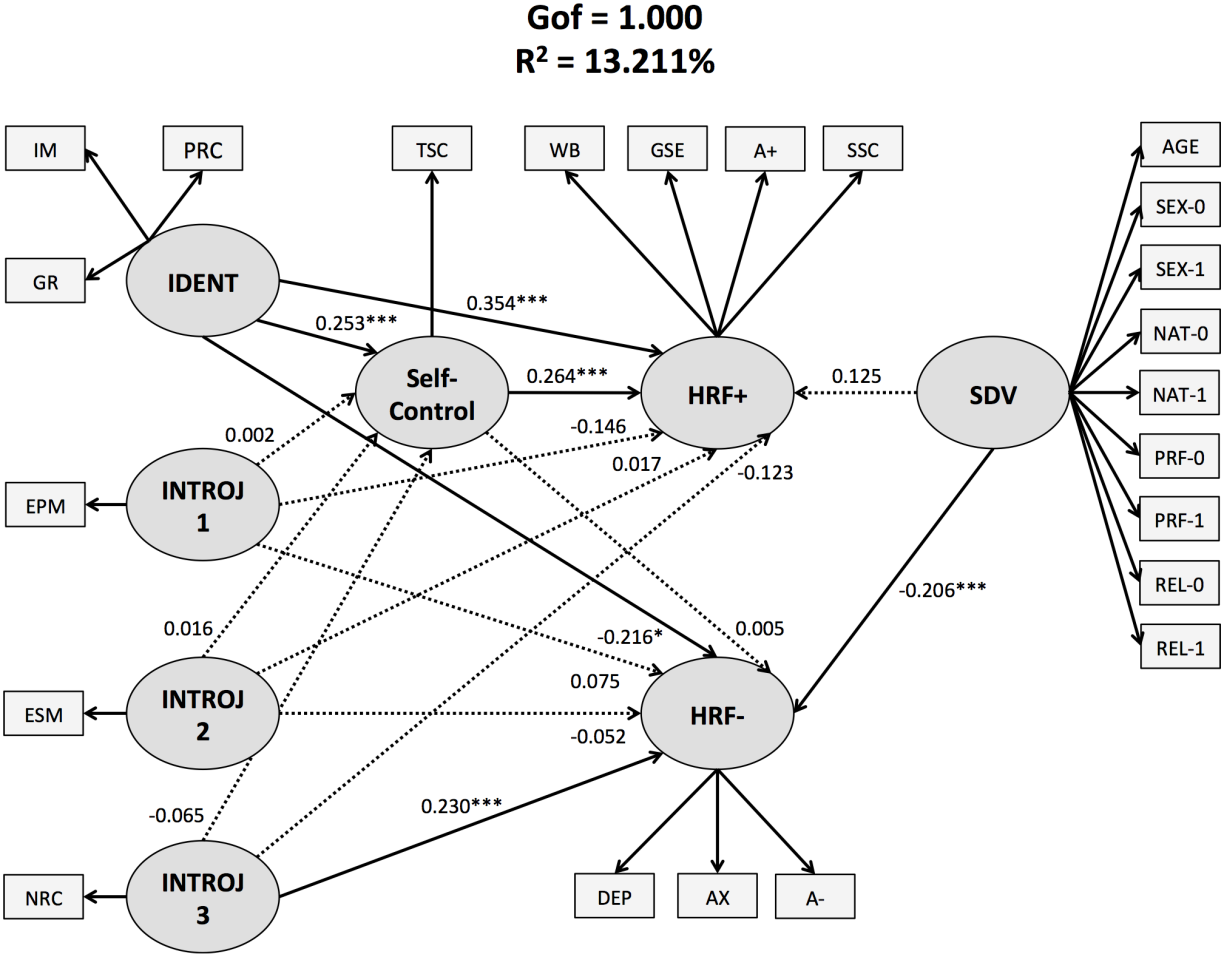
Structural equation model of the relationships among religiosity, self-control (as mediator), and health-related feelings (while controlling for socio-demographic variables). All coefficients are standardized and solid lines indicate statistical significance. **Abbreviations:** TSC = trait self-control; IDENT = identified religiosity; IM = intrinsic motivation; GR = global religiousness; PRC = positive religious coping; INTROJ = introjected religiosity; EPM = extrinsic personal religious motivation; ESM = extrinsic social religious motivation; NRC = negative religious coping; HRF+ = positive health-related feelings; A+ = positive affect; SSC = state self-control; GSE = general self-esteem; WB = wellbeing; HRF- = negative health-related feelings; A- = negative affect; DEP = depression; AX = anxiety; SDV = socio-demographic variables; PRF = professional status (0 = “not working”; 1 = “working”); SEX = gender (0 = “female”; 1 = “male)”; NAT = nation type (0 = “non-Muslim country”; 1 = “Muslim country”); REL = relationship status (0 = “without a partner”; 1 = “with a partner”).

**Fig 2:**
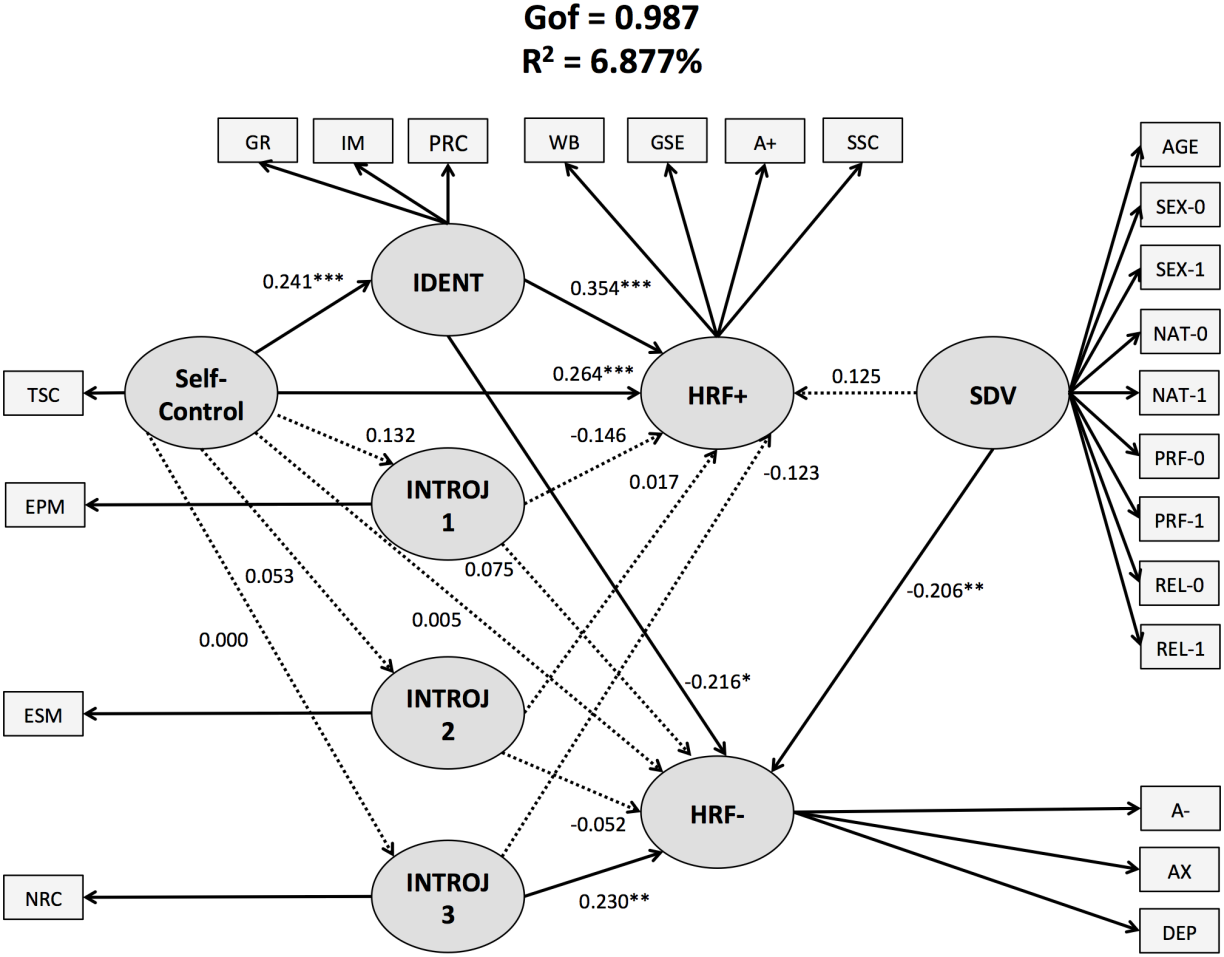
Structural equation model of the relationships among self-control, religiosity (as mediator), and health-related feelings (while controlling for socio-demographic variables). All coefficients are standardized and solid lines indicate statistical significance. **Abbreviations:** TSC = trait self-control; IDENT = identified religiosity; IM = intrinsic motivation; GR = global religiousness; PRC = positive religious coping; INTROJ = introjected religiosity; EPM = extrinsic personal religious motivation; ESM = extrinsic social religious motivation; NRC = negative religious coping; HRF+ = positive health-related feelings; A+ = positive affect; SSC = state self-control; GSE = general self-esteem; WB = wellbeing; HRF- = negative health-related feelings; A- = negative affect; DEP = depression; AX = anxiety; SDV = socio-demographic variables; PRF = professional status (0 = “not working”; 1 = “working”); SEX = gender (0 = “female”; 1 = “male)”; NAT = nation type (0 = “non-Muslim country”; 1 = “Muslim country”); REL = relationship status (0 = “without a partner”; 1 = “with a partner”).

To examine our mediation hypotheses, zero-order correlations between all variables of interest (see Table 1) and SEM analyses were conducted (see Tables 2 and 3, and Figs 1 and 2). SEM analysis consists in building and testing causal models [37]. It combines a measurement model that uses latent variables (LVs), corresponding to dimensions that are observed through a panel of observable indicators called manifest variables (MVs), and a structural model that relates LVs together. The analyses were computed by using the Partial Least Squares (PLS) approach to SEM, also called PLS Path Modeling (PLS-PM).

**Table 1:** Zero-order correlations for all variables of interest.

Variable Name	1	2	3	4	5	6	7	8	9	10	11	12	13
1. GR													
2. IM	.68***												
3. EPM	.44***	.52***											
4. ESM	.10	.21**	.21**										
5. PRC	.64***	.74***	.67***	.11									
6. NRC	.15*	.21**	.33***	.10	.35***								
7. TSC	.23**	.22**	.14	.09	.14	.00							
8. WB	.34***	.31***	.04	.09	.27***	-.04	.25***						
9. GSE	.24**	.11	.01	.10	.06	-.09	.25***	.47***					
10. A+	.23**	.16*	.03	-.01	.11	-.07	.28***	.34***	.43***				
11. SSC	.13	.07	.00	-.06	.05	-.05	.17*	.37***	.48***	.66***			
12. AX	-.14	-.08	-.03	-.10	-.11	.07	-.05	-.37***	-.32***	-.18*	-.37***		
13. DEP	-.08	.02	-.06	-.04	-.01	.22**	.04	-.21**	-.18**	-.17*	-.37***	.58***	
14. A-	-.11	-.09	.08	-.04	-.06	.26***	-.10	-.37***	-.35***	-.21**	-.45***	.72***	.64***

Significance thresholds are

*p* <.001 = ***,

*p* <.01 = **, and

*p* <.05 = *.

GR = global religiousness; IM = intrinsic motivation; EPM = extrinsic personal motivation; ESM = extrinsic social motivation; PRC = positive religious coping; NRC = negative religious coping; TSC = trait self-control; WB = wellbeing; GSE = general self-esteem; A+ = positive affect; SSC = state self-control; AX = anxiety; DEP = depression; A- = negative affect.

**Table 2:** Unidimensionality of MVs blocks.

LV Name	# of MVs	Cronbach’s	D.G.’s *ρ*	PCA eigenvalues
Identified religiosity	3	0.883	0.928	2.434
				0.339
				0.227
Introjected religiosity	3	0.451	0.732	1.442
				0.904
				0.654
HRF+	4	0.784	0.861	2.436
				0.761
				0.494
				0.309
HRF-	3	0.854	0.912	2.325
				0.419
				0.256

LV = latent variable; MV = manifest variable; D.G.’s *ρ* = Dillon-Goldstein’s rho; PCA = principal component analysis; HRF+ = positive health-related feelings; HRF- = negative health-related feelings.

**Table 3:** Factor analyses of MVs blocks.

MV Name	Identified religiosity	Introjected religiosity 1	Introjected religiosity 2	Introjected religiosity 3	Self-Control	HRF+	HRF-
IM	**0.918**	0.533	0.221	0.215	0.234	0.253	-0.053
GR	**0.905**	0.427	0.114	0.158	0.231	0.316	-0.124
PRC	**0.873**	0.661	0.127	0.335	0.174	0.177	-0.059
EPM	0.581	**1.000**	0.224	0.319	0.132	0.041	0.055
ESM	0.172	0.224	**1.000**	0.103	0.053	0.052	-0.060
NRC	0.247	0.319	0.103	**1.000**	0.000	-0.084	0.229
TSC	0.241	0.132	0.053	0.000	**1.000**	0.339	-0.046
A+	0.206	0.037	-0.017	-0.079	0.319	**0.800**	-0.198
SSC	0.109	0.003	-0.058	-0.052	0.205	**0.781**	-0.452
GSE	0.159	0.024	0.110	-0.080	0.226	**0.767**	-0.340
WB	0.349	0.049	0.099	-0.048	0.268	**0.753**	-0.359
A-	-0.120	0.065	-0.049	0.246	-0.099	-0.429	**0.917**
DEP	-0.020	0.071	-0.031	0.230	0.021	-0.293	**0.881**
AX	-0.119	-0.020	-0.101	0.074	-0.047	-0.393	**0.830**

MV = manifest variable; HRF+ = positive health-related feelings; HRF- = negative health-related feelings; IM = intrinsic motivation; GR = global religiousness; PRC = positive religious coping; EPM = extrinsic personal motivation; ESM = extrinsic social motivation; NRC = negative religious coping; TSC = trait self-control; A+ = positive affect; SSC = state self-control; GSE = general self-esteem; WB = wellbeing; A- = negative affect; DEP = depression; AX = anxiety.

#### Dimensionality of LVs

Several tools were used for checking the unidimensionality of the LVs. Cronbach’s alpha and Dillon-Goldstein’s rho were used to determine the composite reliability of the LVs. A block of MVs was considered as unidimensional when both indexes were above 0.7. Principal component analysis with factorial loading was also used to check the unidimensionality of the LVs. A block of MVs was considered as unidimensional when the first eigenvalue of the correlation matrix was higher than 1, while the other eigenvalues were smaller.

#### Path coefficients

EM provides numerical estimates (i.e., path coefficients) to indicate the strength of the causal links of the competing structural models. The quality of each model was assessed with a non-parametric measurement, called goodness of fit (Gof) [37]. The data were controlled for socio-demographic variables (i.e., age, sex, nation, professional status, and relationship status) as potential confounders. PLS-PM was carried out with ordinary least squares. Path coefficients were reported after standardization.

A direct pathway corresponds to the link connecting the input variable and the output variable, while the indirect pathways corresponds to the links between the input variable and the mediator, and between the mediator and the output variable. In the starting (alternative) model, the input variable was religiosity (TSC), while the mediator was TSC (religiosity). In both models, the output variable was HRF. There was a mediating effect when the indirect pathways were significant. When the direct pathway connecting the input variable and HRF was not significant, so that the input variable had no direct effect on HRF, full mediation was found. Conversely, when the direct pathway was significant, a partial mediation was found.

## Results

### Correlations

Zero-order correlations revealed several findings (see Table 1). First, the variables related to the notion of identified religiosity were all highly related to each other (*r*’s = .64 to .74). However, the variables related to the notion of introjected religiosity displayed inconsistent findings: While extrinsic personal motivation was positively related to extrinsic social motivation (*r* = .21) and to negative religious coping (*r* = .33), extrinsic social motivation appeared to be unrelated to negative religious coping (*r* = .10). Second, TSC and identified religiosity were both positively related to wellbeing (*r*’s = .25 to .34), while they were unrelated to negative HFR. Among the variables related to the notion of introjected religiosity, only negative religious coping was positively related to negative HRF, such as anxiety (*r* = .26) and depression (*r* = .22). Third, positive HRF (*r*’s = .34 to .66), as well as negative HRF (*r*’s = .58 to .72), were all positively related to each other. Positive HRF were negatively related to negative HRF (*r*’s = -.17 to-.45).

### Structural Equation Model

#### Dimensionality of LVs

The factor analysis yielded three factors with eigenvalues greater than 1: identified religiosity (3 MVs), positive HRF (4 MVs), and negative HRF (3 MVs) (see Tables 2 and 3). Introjected religiosity appeared multidimensional, with three distinct introjected religiosity patterns: extrinsic personal religious motivation (introjection 1), extrinsic social religious motivation (introjection 2), and negative religious coping (introjection 3) (see Tables 2 and 3).

#### Competing Models

The analyses revealed that the starting model explained 13.211% of the variance and had a Gof of 1.000 (see Fig 1). TSC appeared to partially mediate the relationship between identified religiosity and positive HRF (see Fig 1). Specifically, the direct link connecting identified religiosity and positive HRF yielded a path coefficient of 0.354 ± 0.083 (t = 4.281, p < 0.001). The indirect link connecting identified religiosity and TSC yielded a value of 0.253 ± 0.088 (t = 2.882, p = 0.004), while the indirect link between TSC and positive HRF yielded a value of 0.264 ± 0.068 (t = 3.899, p < 0.001).

The analyses also revealed that the alternative model explained 6.877% of the variance and had a Gof of 0.987 (see Fig 2). First, identified religiosity partially mediated the relationship between TSC and positive HRF (see Fig 2). The direct link connecting TSC and positive HRF yielded a path coefficient of 0.264 ± 0.068 (t = 3.899, p < 0.001). The indirect link between TSC and identified religiosity yielded a value of 0.241 ± 0.071 (t = 3.407, p = 0.001), while the indirect link between identified religiosity and positive HRF yielded a value of 0.354 ± 0.083 (t = 4.281, p < 0.001).

Second, identified religiosity appeared to fully mediate the relationship between TSC and negative HRF (see Fig 2). The direct link between TSC and negative HRF was not statistically significant (t = -0.072, p = 0.943), with a value of -0.005 ± 0.072, while the indirect links between TSC and identified religiosity (0.241 ± 0.071, t = 3.407, p = 0.001) and between identified religiosity and negative HRF (-0.216 ± 0.087, t = -2.471, p = 0.014) proved to be statistically significant.

## Discussion

The present study attempted to test the hypothesis that TSC mediates the relationship between religiosity and HRF in healthy Muslims. This study supported this hypothesis by showing that TSC appeared to partially mediate the relationship between identified religiosity and positive HRF (see Fig 1). As a result, this finding also supports the general view that religion may foster psychosocial outcomes through enhanced self-monitoring and self-control [1, 20]. Moreover, TSC did not mediate the link between religiosity and negative HRF, running counter previous findings that have shown that self-control mediated the link between global religiousness and unhealthy behaviors (see [1, 21–23]). However, consistent with studies that have reported that self-control was unrelated to extrinsic religiosity [15, 38], our findings revealed that TSC was unrelated to the different patterns of introjected religiosity (i.e., extrinsic personal motivation, extrinsic social motivation, and negative religious coping). Taken together, these findings suggest that self-regulation of affects would particularly involve identified religiosity in religious people.

To test the effectiveness of the starting model (Fig 1), which used TSC as mediator of the relationship between religiosity and HRF, it was compared with an alternative model using religiosity as mediator of the TSC-HRF relationship (Fig 2). Firstly, the analyses of the alternative model showed that identified religiosity fully mediated the relationship between TSC and negative HRF (Fig 2), suggesting that TSC may have beneficial effects on negative HRF through enhanced identified religiosity. This finding also indicates that both models predicted HRF, supporting the view that the religion’s links with psychosocial outcomes may reflect bidirectional relationships between religiosity and self-control [39]. Secondly, the comparison of both models revealed that the starting model explained a greater amount of variance than the alternative model (*starting model*: 13.211% *vs*. *alternative model*: 6.877%). This suggests that the hypothesis that TSC mediates the relationship between religiosity and HRF is stronger than the competing hypothesis.

We would hasten to point out that the present study is not without its limitations. Firstly, we used a sample including only Muslims. As a result, caution should be taken when generalizing the present results to the whole religious people, and further studies should examine whether and how psychosocial benefits of religion explained by TSC can be found with other religions (e.g., Judaism, Christianity, and Buddhism). Secondly, this study was based on correlations, and stronger causal tests would be required to examine the hypothesis that religiosity predicts HRF through self-control. In so doing, experimental studies should examine the effects of the activation of concepts and themes related to identified or introjected religiosity on self-control and affects, thereby allowing examining the mediating role of self-control in the relationship between religiosity and affects. Moreover, by considering religiousness as a developmental process, longitudinal studies should examine how religiosity, self-control, and affects fluctuate over the life. This research direction should not only explore how these variables are interconnected within long-term changes, but also shed the light on the mediating mechanisms underlying affects changes in religious people.

From an applied perspective, given that personality can be shaped by religious education [17], teachers should promote identified religiosity. To do so, they should support people’s (e.g., children) fundamental needs for competence (e.g., providing supportive feed-back, inciting focus on internal rewards), autonomy (e.g., being self-direction supportive), and relatedness (e.g., providing a secure attachment), because these factors represent principal sources of self-determination (e.g., [40]). Additionally, conveying positive God conceptions, such as viewing God as a protector in one’s life, should incite people to internalize an agreeable and supportive conception of God, thereby leading to reinforce identified religiosity, which would shape a strong sense of general wellbeing, happiness, and self-control among religious people. Further studies should test the influence of religious educational program based on identified religiosity on the development of self-control, affects, social and healthy behaviors.

## Conclusion

The present study represents the first temptation consisting in testing the hypothesis that TSC mediates the relationships between identified-introjected religiosity and HRF, and evidenced this hypothesis by reporting that TSC mediated the link between identified religiosity and HRF. This study also evidenced that identified religiosity mediated the relationship between TSC and negative HRF. Thus, this study also evidenced that the relationship between religiosity, self-control and HRF is by nature complex.
